# Single and Combined Associations of Plasma and Urine Essential Trace Elements (Zn, Cu, Se, and Mn) with Cardiovascular Risk Factors in a Mediterranean Population

**DOI:** 10.3390/antiox11101991

**Published:** 2022-10-07

**Authors:** Rocío Barragán, Cristina Sánchez-González, Pilar Aranda, José V. Sorlí, Eva M. Asensio, Olga Portolés, Carolina Ortega-Azorín, Laura V. Villamil, Oscar Coltell, Juan Llopis, Lorenzo Rivas-García, Dolores Corella

**Affiliations:** 1 Department of Preventive Medicine and Public Health, School of Medicine, University of Valencia, 46010 Valencia, Spain; 2 CIBER Fisiopatología de la Obesidad y Nutrición, Instituto de Salud Carlos III, 28029 Madrid, Spain; 3 Sport and Health Research Centre, University of Granada, 18016 Granada, Spain; 4 Department of Physiology, Institute of Nutrition and Food Technology ‘‘José Mataix”, Biomedical Research Centre, 18100 Granada, Spain; 5 Department of Computer Languages and Systems, Universitat Jaume I, 12071 Castellón, Spain; 6 Department of Physiology, School of Medicine, University Antonio Nariño, 111511 Bogotá, Colombia

**Keywords:** zinc, copper, selenium, manganese, cardiovascular risk factors, mixture, quantile-g-computation

## Abstract

Trace elements are micronutrients that are required in very small quantities through diet but are crucial for the prevention of acute and chronic diseases. Despite the fact that initial studies demonstrated inverse associations between some of the most important essential trace elements (Zn, Cu, Se, and Mn) and cardiovascular disease, several recent studies have reported a direct association with cardiovascular risk factors due to the fact that these elements can act as both antioxidants and pro-oxidants, depending on several factors. This study aims to investigate the association between plasma and urine concentrations of trace elements and cardiovascular risk factors in a general population from the Mediterranean region, including 484 men and women aged 18–80 years and considering trace elements individually and as joint exposure. Zn, Cu, Se, and Mn were determined in plasma and urine using an inductively coupled plasma mass spectrometer (ICP-MS). Single and combined analysis of trace elements with plasma lipid, blood pressure, diabetes, and anthropometric variables was undertaken. Principal component analysis, quantile-based g-computation, and calculation of trace element risk scores (TERS) were used for the combined analyses. Models were adjusted for covariates. In single trace element models, we found statistically significant associations between plasma Se and increased total cholesterol and systolic blood pressure; plasma Cu and increased triglycerides and body mass index; and urine Zn and increased glucose. Moreover, in the joint exposure analysis using quantile g-computation and TERS, the combined plasma levels of Zn, Cu, Se (directly), and Mn (inversely) were strongly associated with hypercholesterolemia (OR: 2.03; 95%CI: 1.37–2.99; *p* < 0.001 per quartile increase in the g-computation approach). The analysis of urine mixtures revealed a significant relationship with both fasting glucose and diabetes (OR: 1.91; 95%CI: 1.01–3.04; *p* = 0.046). In conclusion, in this Mediterranean population, the combined effect of higher plasma trace element levels (primarily Se, Cu, and Zn) was directly associated with elevated plasma lipids, whereas the mixture effect in urine was primarily associated with plasma glucose. Both parameters are relevant cardiovascular risk factors, and increased trace element exposures should be considered with caution.

## 1. Introduction

Essential trace elements, also known as trace minerals, are micronutrients that are needed in very small amounts through diet but are critical for the prevention of acute and chronic diseases [1,2]. Furthermore, because each essential trace element is linked to multiple enzymes, deficiency of one of these elements can contribute to a variety of metabolic abnormalities and clinical conditions such as diabetes, metabolic syndrome, and cardiovascular diseases, among many others [1,2,3,4,5,6,7]. Even though recent research has demonstrated the significance of the essential trace element in common diseases, nutritional advice has focused on deficiencies that can occur in both acute and chronic diseases and knowledge on the potential risk effects of increased dietary intakes or supplements still remains limited [5,6,7,8,9,10,11,12,13]. Essential trace element classification varies slightly depending on the criteria considered [1,2,3,4]. The World Health Organization classified zinc (Zn), copper (Cu), selenium (Se), manganese (Mn), chromium (Cr), cobalt (Co), iodine (I), and molybdenum (Mo) as essential trace elements in 1973 [2]. Other elements, such as iron (Fe) and boron (B), were later added to this classification (Frieden’s Classification in 1981) [2]. In this study, we will focus on four essential trace elements that are frequently investigated for their relationship with cardiovascular risk factors (Zn, Cu, Se, and Mn). Having a significant function as dietary antioxidant micronutrients, these trace elements are cofactors in a large number of enzymes that participate in the antioxidant defense system and are related to changes in the body’s homeostatic mechanisms, especially inflammation and oxidative stress, which are vital for optimum health [14,15,16,17,18].

In addition to foods, these trace elements can also be obtained from drinking water and environmental/occupational exposures [19,20,21,22,23]. However, it has been estimated that in the general population, diet is the primary factor strongly influencing daily intake of these essential elements [24,25]. Meat and meat products have been reported as the main dietary source of Zn exposure in many countries [20,26,27,28,29,30]. Other items supplying greater Zn in Western diets include cereals, milk and dairy products, nuts, and seeds [31,32]. Cereals, followed by fresh fruits and vegetables, were considered to be the main sources of Cu in Mediterranean subjects [32,33,34]. In other populations, fruits, nuts, and cereals, in addition to tap water have been informed as good sources of dietary Cu [30,35]. Cereals and meat are the leading sources of Se intake in Mediterranean and Western populations, followed by fish, seafood, organ meats, nuts, milk, and dairy products [31,32,33,34,36]. Foods richest in Mn are nuts and seeds, cereals, seafood, legumes, fruits, chocolate, coffee, and tea [34,37,38,39]. However, in addition to the trace elements naturally present in foods, it has been reported that the use of multimineral supplements is the most significant dietary determinant of their intake in certain populations [40,41]. Due to public perceptions that the deficiency of certain minerals is linked to diseases, the usage of multivitamin/multimineral supplements has expanded over the past few decades [42,43]. The initial studies demonstrating the important antioxidant properties of the trace elements Zn, Cu, Se, and Mn [44,45,46,47,48] as well as the links between deficiencies of these trace elements and several diseases [49,50,51,52,53,54], contributed to this perception.

However, high levels of these trace elements may have adverse effects, including toxicity [55,56,57,58]. Thus, caution is required when increasing their intakes given the harmful effects when present in quantities exceeding those physiologically necessary. Thus, Zn acts as a pro-oxidant at a range of concentrations [59]. Zn excess as well as deficiency are pro-oxidant conditions. High concentrations of Zn have been linked to zinc inhibition of antioxidant enzymes, which result in increased reactive oxygen species formation. The thresholds determining the Zn concentrations that affect its function as a pro-antioxidant and a pro-oxidant have not been properly established [60]. Excessive Cu exposure tends to result in the overproduction of reactive oxygen species, which can cause oxidative-stress-induced cellular damage [61]. High blood selenium levels can lead to selenosis [62]. High Mn levels have been associated with increased oxidative stress and induced neurodegeneration [63,64]. The ability of Mn to enhance oxidative stress is due to the transition of its oxidative state +2 to +3, which increases its pro-oxidant capacity [65]. However, the current emphasis is on the chronic effect of moderately high concentrations of these elements, given the growing evidence of an increased risk of cardiometabolic diseases such as diabetes, hypertension, dyslipidemias, and some cardiovascular outcomes [66,67,68,69,70,71,72,73,74,75,76,77]. Despite the large number of studies [66,67,68,69,70,71,72,73,74,75,76] that show a direct relationship between Se, Cu, Zn, or Mn and cardiovascular risk factors, there are other studies that show no associations or even an inverse relationship [51,78,79,80,81,82,83,84]. Many factors can contribute to the disparities in the findings of various studies. The most important are population characteristics (sex, age, geographical origin, diet, pathologies, or even genetic factors), as well as the measurement used to assess trace element levels. Several investigations have been conducted to assess the amount of trace elements provided by the diet [3,21,27,29,30,31,32,33,34,35,36,37,38,39,71,78,81]. However, it is well known that the content of trace elements in food varies greatly depending on the composition of the soil, water, and the environment [2]. As a result, it is preferable to use other, more objective measures of Zn, Cu, Se, and Mn status, such as analyzing their concentrations in biological samples [85,86,87,88,89,90,91]. For each trace element, there are different types of biomarkers in blood, plasma, urine, hair, adipose tissue, and nails. Each has its own set of advantages and disadvantages [86,87,88,89,90,91,92,93,94]. Plasma/serum concentrations are the most commonly used, though there may be differences between studies.

Therefore, in the new era of personalized nutrition, greater emphasis must be placed on the characteristics of the population analyzed as well as on the proper interpretation of the biomarkers used in each study [95]. The majority of published studies have concentrated on trace element analyses separately. However, the significance of analyzing several combined trace elements in what is known as “mixture” analysis has been emphasized because the concentration of one element can influence the effects of the other [96,97,98,99,100]. Accordingly, current recommendations insist on conducting these combined analyses using novel statistical methodologies [101,102,103]. With this context in mind, our goals are as follows: (1) To investigate the single associations between trace element concentrations in plasma and the main cardiovascular risk factors in a general Mediterranean population. (2) To analyze these single associations in urine. (3) To conduct combined analyses of the associations between trace elements and cardiovascular risk factors in this population using three approaches—principal components analysis, quantile-based g-computation, and the calculation of so-called trace element risk scores (TERS).

## 2. Materials and Methods

### 2.1. Study Design and Participants

We conducted a cross-sectional analysis on 484 Caucasian participants in the OBENUTIC-Mineral study [104], a sub-study consisting of 500 persons preselected from the OBENUTIC study. OBENUTIC stands for Obesity, Nutrition, and Information and Communication Technologies. It is an open case-control study of the general population of Valencia, Spain (consisting of men and women aged 18 to 80), without sex and age pairing [105]. Cases were obese subjects (body mass index (BMI) ≥ 30 kg/m^2^) and the controls were unpaired non-obese individuals recruited from the same region. Pregnancy or breastfeeding, invalidating physical or psychological disorders, cancer diagnosis, thyroid changes, Cushing disease, suffering from infectious/contagious disease, excessive alcohol use, or use of other drugs were exclusion factors. Focusing on the OBENUTIC-Mineral study [104], we preselected a sub-set of 500 individuals who were consecutively recruited in the OBENUTIC study over a 22-month period. A total 492 participants provided enough biological samples (plasma and urine) for the trace element determinations. We found 8 samples with extreme values that differed from other observations, indicating potential measurement errors or other incidents related to sample handling. These samples were deemed outliers and were thus excluded. Therefore, 484 participants were included for statistical analysis. The investigation was conducted at the Department of Preventive Medicine and Public Health, School of Medicine, University of Valencia, Valencia. Participants provided written informed consent, and the protocol and methods were approved by the Human Research Ethics Committee of the University of Valencia, Valencia (reference number: H1488282121722; 06/04/2017).

### 2.2. Demographic, Anthropometric, Biochemical, Clinical, and Lifestyle Variables

A standardized questionnaire previously used in our studies [105,106] was employed to collect socio-demographic information, clinical variables, medication use, and lifestyle variables. According to the World Health Organization, a current smoker was considered as someone who smokes any tobacco product at least once a day. Non-smokers included both never smokers and former smokers [104]. A validated 14-item scale was used to assess adherence to the Mediterranean diet [107]. Based on our previous results [107], subjects were classified as having low Mediterranean diet adherence (less than 9 points) or high adherence (9 or more points). Participants’ heights were measured using a standard stadiometer built into the scales (SECA Mod 220. Seca Deutschland Gmbh and Co. Kg., Hamburg, Germany). Calibrated scales (TANITA-BC-420-S, Tanita UK Ltd., Middlesex, UK) were used to determine weight [104]. BMI was calculated by dividing weight in kilograms by height in meters squared. Obesity was defined as having a BMI greater than 30 kg/m^2^. Using an anthropometric tape, the waist circumference was measured halfway between the lowest rib and the iliac crest. Waist circumference of 102 cm in men or 88 cm in women was considered high. An automatic sphygmomanometer was used to measure systolic and diastolic blood pressures (Omron HEM-705CP, OMRON Healthcare Europe B.V., Hoofddorp, The Netherlands). Hypertension was defined as having a systolic blood pressure of 140 mmHg or a diastolic blood pressure of 90 mmHg or being on antihypertensive medication.

After a 12 h overnight fast, blood samples were collected. Centrifugation was used to obtain plasma samples, and standard biochemical analyses were performed the same day. Furthermore, plasma samples were kept at −80 °C for future analyses (i.e., Zn, Cu, Se, and Mn determinations). In a certified clinical laboratory, fasting plasma glucose, total cholesterol, HDL-cholesterol, and triglyceride concentrations were determined using previously described enzymatic methods [104] (Olympus AU5400. Beckman Coulter, CA, USA), and LDL-cholesterol was estimated using the Friedewald equation. Plasma creatinine was tested with the Jaffé method, uric acid was determined with the uricase method, and aspartate aminotransferase was assessed with a standard method implemented on a multi-autoanalyzer manufactured by Roche Diagnostics (Basel, Switzerland). Diabetes was defined as having a fasting glucose level of 126 mg/dL or being on diabetes medication). For hypercholesterolemia, we used total cholesterol and LDL-cholesterol. First, we defined total hypercholesterolemia as having total cholesterol levels ≥ 200 mg/dL or taking lipid-lowering drugs. Second, we defined high LDL cholesterol levels as LDL ≥ 160 mg/dL or taking lipid-lowering medications. In addition, a first voided urine sample was collected on the same day as the blood sample and stored at −80 °C for later analysis.

### 2.3. Zn, Cu, Se, and Mn Determinations

Determination of Zn, Cu, Se, and Mn total content in plasma and urine samples was performed using an inductively coupled plasma mass spectrometer (ICP-MS) (Agilent 7500. Agilent Technologies, Tokyo, Japan) fitted with a Meinhard type nebulizer (Glass Expansion, Romainmotier, Switzerland) and equipped with a He collision cell. A Milli-Q system (Millipore, Bedford, MA, USA) was used to obtain deionized water (18 MΩ). All reagents (Ammonium Hydroxide Solution, Butanol, EDTA, Triton X-100, NHO_3_, and HCl) used were of the highest available purity. A standard solution of 100 µg/L of Li, Mg, Sc, Co, Y, In, Ce, Ba, Pb, Bi, and U in 1% (*v*/*v*) HNO_3_ was prepared from a 1.000 mg/dL multi-element stock standard solution (Merck & Co. Inc., Whitehouse Station, NJ, USA) and used for daily optimizing of the ICP parameters as previously described [104]. Single-element standard solution for ICP-MS containing 1.000 µg/mL of Cu, Zn, Mn, and Se (Merck & Co. Inc., Whitehouse Station, NJ, USA). The plasma samples were previously prepared with a basic solution containing ammonium hydroxide, butanol, EDTA, and triton X-100 [108,109]. Urine samples were previously prepared with an acidic solution containing 1% NHO_3_ and 0.5% HCl. Calibration curves were prepared using Ga as an internal standard and by the dilution of stock solutions of 1.000 mg/dL in 1% HNO_3_. The accuracy of this method was evaluated by comparison with certified reference materials Seronorm™ Trace Elements Serum and Seronorm™ Trace Elements Urine (Billingstad, Norway) and by recovery studies of spiked samples with multi-element standards. The calculated recovery was between 95% and 105% in all cases. We used the mean of five separate determinations.

### 2.4. Statistical Analysis for Individual and Combined Associations

We examined the normality of the continuous variables and performed natural logarithmic transformation on all the essential trace element variables determined in plasma and in urine. The triglyceride variable in plasma was also logarithmically transformed for statistical testing. Descriptive analyses for socio-demographic, anthropometric, clinical, biochemical, and lifestyle data were carried out. To compare means in continuous variables, the T-test and ANOVA analysis were used. The Spearman correlation test was used to examine correlations between trace element levels in plasma and urine. First, we analyzed the association between each plasma/urine trace element and the corresponding cardiovascular risk factor individually. As cardiovascular risk factors, we considered plasma lipid concentrations (total cholesterol, LDL-cholesterol, HDL-cholesterol and triglycerides); blood pressure (SBP and DBP); and fasting plasma glucose, BMI, and waist circumference (all of them as continuous variables). In addition, we analyzed some cardiovascular risk factors as categorical variables (sex, age groups, hypertension, diabetes, hypercholesterolemia, and high waist circumference) using the cut-off points previously defined. We fitted generalized linear models to examine the individual relationships between plasma or urine trace elements (Zn, Cu, Se, and Mn) and cardiovascular risk factors as continuous variables. We used logistic regression models to estimate the odds ratios (OR) and 95% confidence intervals (CI) associated with categorical variables as cardiovascular risk factors. For both the linear and logistic models, potential confounders were adjusted sequentially as follows: Model 1, unadjusted model; Model 2, model adjusted for age and sex; Model 3, model adjusted for age, sex, obesity, and medications (lipid-lowering, antihypertensive, or hypoglycemic drugs) when appropriate. When indicated, additional adjustments for smoking and adherence to the Mediterranean diet were made.

Second, we explored the combined association between trace elements and cardiovascular risk factors. As there is currently no consensus on the optimal statistical method for mixtures [99,102], we used 3 approaches to compare results: (a) principal component analysis, (b) quantile-based g-computation, and (c) calculation of the so-called TERS. (a) We performed a factor analysis on the plasma or/and urine trace elements (Zn, Cu, Se, Mn) to determine the latent multidimensionality by identifying a potentially smaller number of unobserved (latent) variables termed factors. We calculated the Kaiser–Meyer–Olkin (KMO) value and Bartlett’s test (homogeneity of variance). Utilizing principal component analysis, components were extracted. In the factor analysis, we determined the ideal number of components using the Kaiser criterion (components with eigenvalues greater than 1). We used orthogonal rotation (varimax) to clarify the factors. The varimax method aims to reduce the number of variables with a high loading on a single latent component [110]. For each participant, the scores of the obtained factors were generated, and these scores were subsequently utilized as latent variables of combined trace elements in the corresponding association analyses shown in results. (b) We also applied the new method so-called quantile-based g-computation [103] for the combined analysis of the plasma/urine trace elements to go one step further than the principal component analysis by summarizing the effect of the complex mixture as a global exposure, creating a single score. This can be achieved by the weighted quantile sum (WQS) regression [111]. However, the WGS regression has several limitations [102]. Therefore, we used here the quantile-based g-computation [103], a new modeling technique that builds on WQS regression by integrating its estimation procedure with g-computation but by estimating the parameters of a marginal structural model and overcoming the assumption of unidirectionality of the WQS, in addition to other advantages [103]. Plasma and urine trace elements (Ln transformed) were preprocessed (standardized) by scaling all of the variables (mean = 0; SD = 1). Quartiles were selected for analysis. Positive and negative associations for each trace element were identified. Either linear or logistic models were fitted for continuous or categorical dependent variables (cardiovascular risk factors) taking into account the positive or negative associations. Quantile-based g-computation was implemented using the qgcomp package [103] (version 2.8.6, 2022) with R studio (R Foundation for Statistical Computing, Vienna, Austria). Unadjusted and multivariate adjusted models (Models 1, 2, and 3) were computed, and the regression coefficients and *p*-values were estimated. (c) Lastly, taking into account the complexity of the quantile-based g-computation method, we proposed a simple method known as TERS and based on a similar procedure used to generate the unweighted genetic risk scores [112]. Plasma and urine trace elements were preprocessed for categorical scaling according to their respective tertiles. From the individual trace element models, we computed the directionality of the effect and identified the tertiles associated with an increased risk for cardiovascular risk factors overall. Separate tertiles of Zn, Cu, Se, and Mn plasma and urine were used to generate additive TERS. The highest-risk tertile was assigned a value of 2, while the lowest-risk tertile was assigned a value of 0. The middle tertile was given a score of 1. This variable ranged from 0 (lower risk) to 8 (higher risk), depending on the trace element concentration (low or high) for plasma or urine. Then, general linear or logistic regression models (unadjusted and adjusted for covariates) were fitted for the TERS and cardiovascular risk factors. SPSS Statistics for Windows Ver. 26 was used to analyze the data (IBM Corp., Armonk, NY, USA). All tests were two-tailed and *p*-values < 0.05 were considered statistically significant.

## 3. Results

### 3.1. General Characteristics of the Population

The general demographic, clinical, biochemical, and lifestyle characteristics of the studied population by sex are summarized in Table 1. Likewise, Table 1 shows the plasma and urine concentrations of the trace elements studied (Zn, Cu, Se, and Mn). This sample was recruited from the general Mediterranean population (aged 18 to 80 years) and was relatively healthy.

There were no statistically significant differences by sex (*p* = 0.520) between men and women (a mean age of 46.28 years). Table 1 also shows the mean values of cardiovascular risk factors, such as plasma lipids (total cholesterol, LDL-cholesterol, HDL-cholesterol, and triglycerides), blood pressure (SBP and DBP), fasting glucose, and anthropometric measurements by sex. Diabetes prevalence (5.4%) was low. The prevalence of hypercholesterolemia, obesity, or hypertension was higher. Zn levels were 15.94 ± 3.45 µmol/L in plasma and 5.60 ± 4.26 µmol/L in urine, with both fluids containing more Zn in men (*p* < 0.001). Cu in plasma averaged 25.50 ± 6.70 µmol/L, while in urine it was 0.13 ± 0.07 µmol/L. Women had higher plasmatic Cu levels than men (*p* < 0.001), whereas men had higher urinary Cu levels (*p* = 0.042). Plasma Se concentration was 1.19 ± 0.19 µmol/L and urinary Se concentration was 0.37 ± 0.21 µmol/L, with statistically significant differences by sex only in urine (*p* = 0.01). Finally, plasma Mn levels were 74.86 ± 38.40 nmol/L and urine Mn levels were 7.66 ± 6.30 nmol/L, with no statistically significant differences by sex observed.

Table S1 displays the correlation (Spearman rho coefficients) and *p*-values between Zn, Cu, Se, and Mn levels in plasma and urine. Zn levels in plasma were found to be significantly and directly related to Zn levels in urine (rho = 0.232; *p* < 0.001). There were only marginally significant correlations between plasma and urine levels for Cu (rho = 0.089; *p* = 0.052) and Se (rho = 0.082; *p* = 0.071). There was a statistically significant inverse correlation between Mn plasma and urine levels (rho = −0.141; *p* = 0.003).

### 3.2. Individual Associations of Trace Elements in Plasma and in Urine with Cardiovascular Risk Factors

Table 2 shows the associations between Zn, Cu, Se, and Mn plasma levels (considered separately) and cardiovascular risk factors as continuous variables. Three different models were considered (unadjusted, adjusted for sex and age, and additionally adjusted for obesity and medications where appropriate).

No statistically significant associations were found between plasma levels of Zn and cardiovascular risk factors. However, some associations were obtained for the Cu plasma levels. In the model adjusted for all of the covariates, statistically significant direct associations were found between plasma Cu levels and plasma triglyceride concentrations (*p* < 0.001). Additional adjustments for smoking and Mediterranean diet adherence had no effect on the statistical significance of the associations (not shown). Similarly, higher plasma Cu levels were associated with higher BMI and waist circumference (both *p* < 0.05). However, plasma Se had the most significant positive associations with total cholesterol (*p* < 0.001), LDL-cholesterol (*p* < 0.001), and HDL-cholesterol (*p* < 0.001) plasma concentrations, which remained statistically significant after multivariate adjustment in model 3 and after additional adjustment for smoking and adherence to the Mediterranean diet. In addition, Se had a weaker positive association with SBP (*p* = 0.030). Plasma Mn was inversely associated with plasma lipids, SBP, DPB, and anthropometric variables in the unadjusted models. However, after adjusting for covariates, these associations were no longer statistically significant. When we considered cardiovascular risk factors as categorical variables, including sex and age (Table S2), we found that both plasma Zn and Cu showed highly significant differences between men and women, even after adjusting for age and other covariates. However, the plasmatic levels of Se and Mn did not present these differences by sex, with the differences by age being more important for them (*p* < 0.05). Plasma Se showed a highly significant association with hypercholesterolemia (*p* < 0.001), while Cu was associated with an increased risk of abdominal obesity after adjusting for age and sex (*p* = 0.022). Table 3 displays the associations between Zn, Cu, Se, and Mn urine levels (considered separately) and cardiovascular risk factors as continuous variables.

Three different models were considered (unadjusted, adjusted for sex and age, and additionally adjusted for obesity and medications where appropriate). Interestingly, when we use these biomarkers in urine, the associations with the same cardiovascular risk factors differ from those found in plasma. This could explain some of the differences in published studies based on the biomarker used to determine the status of the trace elements of interest. Thus, Zn in urine was significantly associated (*p* = 0.007) with fasting plasma glucose even after multivariable adjustment for covariates. No significant association was detected between plasma Zn and plasma glucose (*p* = 0.602) in the same individuals. Cu in urine was inversely associated with HDL-cholesterol (*p* = 0.026) in the multivariable adjusted model and we did not detect the association with triglycerides and BMI found in plasma. Likewise, Se levels in urine were not associated with the higher total cholesterol or LDL-cholesterol that were strongly observed in plasma. No relevant associations were detected between Mn in urine and the cardiovascular risk factors analyzed. When we consider cardiovascular risk factors as categorical variables, including sex and age (Table S3), we detected differences by sex and age for urinary Zn, Cu, and Se, showing the importance of the adjustment for sex and age when analyzing further associations for these trace elements. The only relevant association in urine was detected between Zn and diabetes, which remained statistically significant even after adjustment for sex, age, obesity, and medications (*p* = 0.036). The corresponding OR in the adjusted model was OR = 3.27; 95%CI: 1.60–6.68, per unit increment of urine Zn concentration (ln units).

### 3.3. Associations between Combined Trace Elements in Plasma and Cardiovascular Risk Factors

To conduct combined analyses of the associations between trace elements in plasma and cardiovascular risk factors, we used three approaches: principal components analysis, quantile-based g-computation, and the calculation of TERS.

#### 3.3.1. Principal Component Analysis for Plasma

A factor analysis of the main components, based on the plasma concentration of Zn, Cu, Se, and Mn was undertaken to better understand the latent structure underlying these trace elements. The KMO measure and the Bartlett test of sphericity reached statistical significance (*p* < 0.001). Following the Kaiser criterion of extracting factors with eigenvalues >1, three factors were extracted (Table 4). The first three factors or principal components (PC) cumulatively accounted for 84.1% of the total variance. PC1 explained 32.9% of the total variance, PC2 explained 25.9%, and PC3 explained 25.3%.

**Figure 1:**
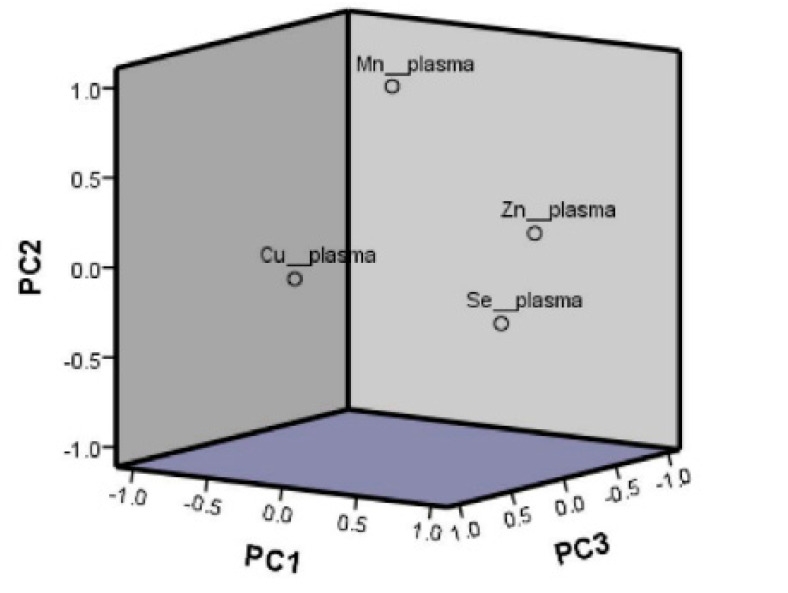
Principal component analysis loading plots for the rotated (varimax rotation) components for the combined plasma trace elements (Zn, Cu, Se, and Mn) in the studied population. PC: principal component.

The PCs may be interpreted as new uncorrelated variables whose characteristics represent those constituent trace elements with the largest loadings. Having undertaken the varimax transformation to better identify the components, we observed (Table 4) that PC1 presented positive high factor loadings with plasma Zn (0.825) and plasma Se (0.791) and can be identified as the component that mainly represented plasma Zn and Se levels. PC2 was heavily loaded with Mn (0.965) whilst PC3 was heavily loaded with Cu (0.988). Figure 1 shows the principal component analysis loading plots for the rotated (varimax rotation) components for this analysis. It can be seen that Mn levels were less associated with the other trace elements. Further, Cu presented a particular association pattern. Therefore, we focused our combined trace element analysis on PC1, mainly representing the combined pattern of plasma Zn and Se. Then, we analyzed the association between PC1 and cardiovascular risk factors. In model 3 adjusted for covariates, PC1 (Zn and Se) was directly and significantly associated with total cholesterol (*p* < 0.002), LDL-cholesterol (*p* = 0.007), HDL-cholesterol (*p* < 0.001), and SBP (*p* = 0.048). No significant associations were found for fasting glucose, DBP, BMI, or waist circumference.

#### 3.3.2. Quantile-Based g-Computation for Plasma

**Figure 2:**
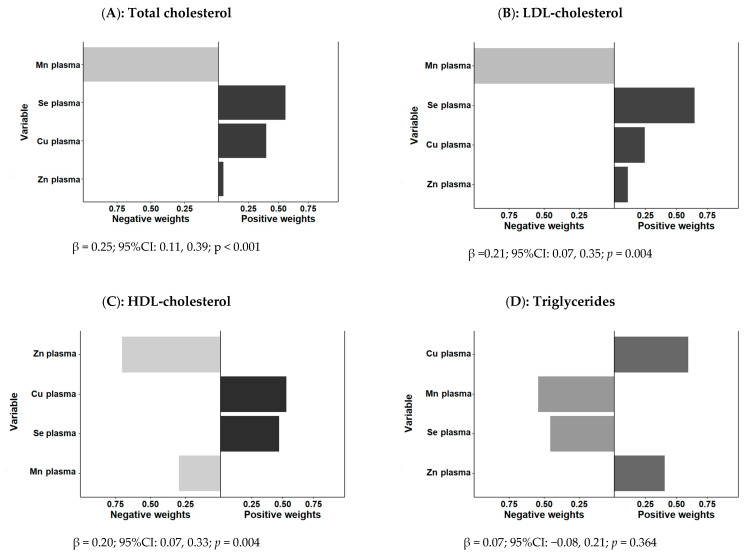
Overall mixture effect estimates and the weights of trace element contributions to the outcome (psi1 beta coefficients, 95% confidence intervals (CI), and *p*-values) for the combined association of plasma Zn, Cu, Se, and Mn with plasma lipid levels: total cholesterol (panel **A**), LDL-cholesterol (panel **B**), HDL-cholesterol (panel **C**), and triglycerides (panel **D**) as continuous variables.

This new approach allows us to estimate the joint effects of the combination (mixture) of plasma Zn, Cu, Se, and Mn on the cardiovascular risk factors. First, we analyzed the cardiovascular risk factors (continuous variables) as separate outcomes. This is a parameter-based, generalized-linear-model-based g calculation implementation to estimate the result change of a quantile while increasing all exposures in a specific mixture [103]. We considered quartiles for all the scaled trace elements as indicated in Methods. We fitted unadjusted models and models sequentially adjusted for sex and age, and additionally adjusted for sex, age, obesity, and medications when applicable (model 3). For the overall exposure, we calculated the so-called “overall mixture effect from quantile g-computation” (psi1). This effect (regression coefficient) is interpreted as the effect on the outcome of increasing every exposure by one quartile, conditional on covariates [103]. g-computation does not require a “directional homogeneity” assumption that all exposures are related to the results in the same direction. This model is achieved by classifying the trace elements into quartiles, coded as 0, 1, 2, and 3, and fitting a linear model. The effect of each trace element can be positive or negative, and depending on this, a weight is given [103]. The estimated quantile g of the exposure response is the sum of the regression coefficients of the included exposures. If the effects of the trace elements have different directions, the weight is interpreted as the positive (or negative) part of the influence in the global estimation. Although we fitted unadjusted and adjusted models, only the results corresponding to the adjusted model 3 are presented. Figure 2 shows the overall mixture effect estimates of trace element contributions to the outcome (psi1 beta coefficients, 95%CIs, and the *p*-values) for the combined association between plasma Zn, Cu, Se, and Mn and plasma lipid levels (as continuous variables). Panels A, B, C, and D show the results for the outcome’s total cholesterol, LDL-cholesterol, HDL-cholesterol, and triglycerides, respectively. Models were adjusted for sex, age, obesity, and medications when appropriate. Figure 2 also shows the weights representing the proportion of the positive or negative partial effect for each trace element in the quantile g-computation model for each outcome variable. The results of the quantile g-computation combined exposure model analysis showed that the combined plasma Zn, Se, Cu, and Mn trace elements were significantly associated with plasma total cholesterol (p<0.001) (panel A), with a combined effect of increasing 0.25 (95%CI: 0.11–0.39) units the outcome per one quartile increase in trace element concentration (z-score normalized). Zn, Se, and Cu presented positive weights, whereas Mn had a negative weight.

These plots are simple to understand when all of the weights are on the same side of the null because the weight corresponds to the proportion of the overall effect from each exposure. However, the weights could go either way, indicating that some exposures are beneficial and others are harmful. Thus, the weights in Figure 2 correspond to the proportion of the overall effect in a particular direction, which may be small (or large) compared to the overall “mixture” effect.

It is critical to remember that the left and right sides of the plot should not be compared because the length of the bars corresponds to the size of the effect only relative to other effects in the same direction. The size of the overall effect is represented by the darkness of the bars; in this case, the bars on the right (positive) side of the plot are darker because the overall “mixture” effect is positive and statistically significant in panels A, B, (beta = 0.21; 95%CI: 0.07–0.35; *p* = 0.004) and C (beta = 0.20; 95%CI: 0.07–0.33; *p* = 0.004), corresponding to total cholesterol, LDL-cholesterol, and HDL-cholesterol). The combined association with plasma triglycerides (panel D) did not reach statistical significance (beta = 0.07; 95%CI: 0.08–0.21; *p* = 0.364). As a result of the shading, one can make informal comparisons between the left and right sides (Figure 2): a large, darkly shaded bar indicates a larger independent effect than a large, lightly shaded bar.

**Figure 3:**
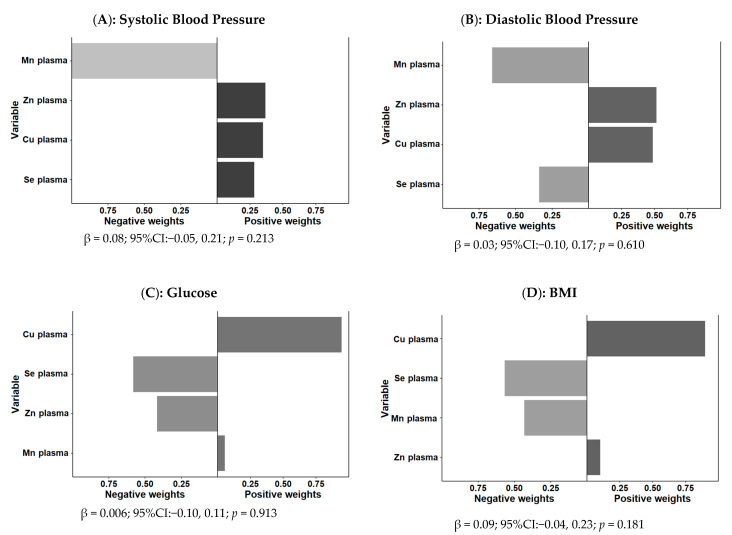
Overall mixture effect estimates and the weights of trace element contributions to the outcome (psi1 beta coefficients, 95% confidence intervals (CI), and *p*-values) for the combined association of plasma Zn, Cu, Se, and Mn with SBP (panel **A**), DBP (panel **B**), fasting glucose (panel **C**), and BMI (panel **D**) as continuous variables. Multivariable adjusted model.

Figure 3 shows the overall mixture effect estimates and the weights of trace element contributions to the outcome (psi1 beta coefficients, 95%CIs, and the *p*-values) for the combined association between plasma Zn, Cu, Se, and Mn and SBP (panel A), DBP (panel B), fasting glucose (panel C), and BMI (panel D) as continuous variables. Models adjusted for sex, age, obesity, and medications. No statistically significant association between the combined mixture and SBP (*p* = 0.213), DBP (*p* = 0.610), fasting glucose (*p* = 0.913), or BMI (*p* = 0.181) were detected. Likewise, when waist circumference was considered as the outcome using the same approach, no statistically significant combined association was found (beta: 0.086; 95%CI: −0.032–0.153; *p* = 0.153).

Next, using the quantile-based g-computation approach, we then assessed cardiovascular risk factors as categorical variables. The joint effect of Zn, Cu, Se, and Mn was associated with an increased probability of having high total cholesterol (OR: 2.03; 95%CI: 1.37–2.99; *p* < 0.001) and high LDL-cholesterol (OR: 2.15; 95%CI: 1.45–3.03; *p* = 0.001). Zn, Cu, and Se all had positive weights, but Mn had negative weights. There were no significant associations seen for hypertension or diabetes.

#### 3.3.3. Calculation of TERS for Plasma

Due to the seeming complexity of the quantile-based g-computation method, we developed a simple method based on additive scores to summarize the combined influence of trace elements, as given in the Methods section. For category scaling, we first determined the tertiles of plasma concentrations for each element (Table 5). On the basis of the individual associations between plasma trace elements and cardiovascular risk factors as continuous variables, we computed the directionality of the effect and determined the tertiles associated with an increased risk for cardiovascular risk factors. For each trace element, the tertile with the highest risk was assigned a value of 2, while the tertile with the lowest risk was assigned a value of 0.

The score for the middle tertile was 1. We built a global additive TERS for plasma (the same TERS for each cardiovascular risk factor) and examined the combined trace element influence on each cardiovascular risk factor. In this TERS, we computed a direct effect increasing cardiovascular risk for Zn, Cu, and Se (scored as 0, 1, and 2 for tertile 1, tertile 2, and tertile 3, respectively). Due to the identified inverse effect for Mn, this trace element was scored inversely (2 for tertile 1, 1 for tertile 2, and 0 for tertile 3). With these scores, the plasma TERS variable for additive combined effect ranged from 0 (lower risk) to 8 (higher risk), depending on the trace element concentration (low or high). Then, unadjusted and adjusted general linear or logistic regression models were built for the TERS and cardiovascular risk variables. Table 6 displays correlation coefficients and *p*-values for the association between the combined score for Zn, Cu, Se, and Mn in plasma and the continuous cardiovascular risk factors.

Models 1, 2, and 3 were fitted. We detected a statistically significant association between the combined plasma TERS and total cholesterol (*p* < 0.001), LDL-cholesterol (*p* < 0.001), and HDL-cholesterol (*p* < 0.001) using model 3 (adjusted for sex, age, obesity, and medications when applicable). There were no statistically significant associations found with glucose at fasting, BMI, waist circumference, and DBP.

These results were comparable to those obtained earlier utilizing the quantile-based g-computation method for plasma. However, we found statistically significant findings for SBP when utilizing the TERS method (*p* = 0.019) but none when utilizing the quantile-based g-computation (*p* = 0.213).

### 3.4. Associations between Combined Urinary Trace Elements and Cardiovascular Risk Factors

Using the same methodology as analyses performed on plasma, we have also investigated the combined effects of trace elements on urine. However, we will not describe the results in such detail as there are more limitations to using trace element concentrations in urine as good biomarkers than in plasma, and the results may be more biased.

#### 3.4.1. Principal Component Analysis for Urine

A factor analysis of the main components based on urine concentration of Zn, Cu, Se, and Mn was undertaken to better understand the latent structure underlying these trace elements. The KMO measure and the Bartlett test of sphericity reached statistical significance (*p* < 0.001). Following the Kaiser criterion of extracting factors with eigenvalues >1 (2.233 and 1.003, respectively), two factors were extracted. The two factors, or PCs, cumulatively accounted for 80.8% of the total variance. PC1 explained 55.8% of the total variance and PC2 explained 25.1%. Having undertaken the varimax transformation to better identify the components, PC1 presented positive high factor loadings with urine Zn (0.811), urine Cu (0.908), and urine Se (0.865) and can be identified as the component that mainly represented the combined Zn, Cu, and Se levels. PC2 was heavily loaded with Mn (0.999), representing this trace element clearly separated from the others. Then, we analyzed the association between PC1 in urine and cardiovascular risk factors. In model 3 adjusted for covariates, PC1 was only significantly and directly associated with fasting glucose (*p* = 0.02). No significant associations were found for plasma lipids, blood pressure, BMI, or waist circumference.

#### 3.4.2. Quantile-Based g-Computation for Urine

We estimated the joint effects of the combination (mixture) of urine Zn, Cu, Se and Mn on cardiovascular risk factors. First, we analyzed the cardiovascular risk factors (continuous variables) as separate outcomes. As stated in Methods, we considered quartiles for all scaled trace elements in urine. We fitted unadjusted models and models sequentially adjusted for sex and age, and additionally adjusted for sex, age, obesity, and medications, when appropriate (model 3). For the overall exposure, we calculated the “overall mixture effect from quantile-based g-computation” (psi1) [103]. Table 7 shows the overall mixture effect estimates of trace elements contributions to the outcome (psi1 beta coefficients, 95%CIs, and the *p*-values) for the combined association between urine Zn, Cu, Se, and Mn and plasma lipid levels (total cholesterol, LDL-cholesterol, HDL-cholesterol, triglycerides, SBP, DBP, BMI, and waist circumference). We only found statistically significant combined associations for plasma glucose (Beta: 0.14; 95%CI: 0.03–0.26; *p* = 0.014).

**Figure 4:**
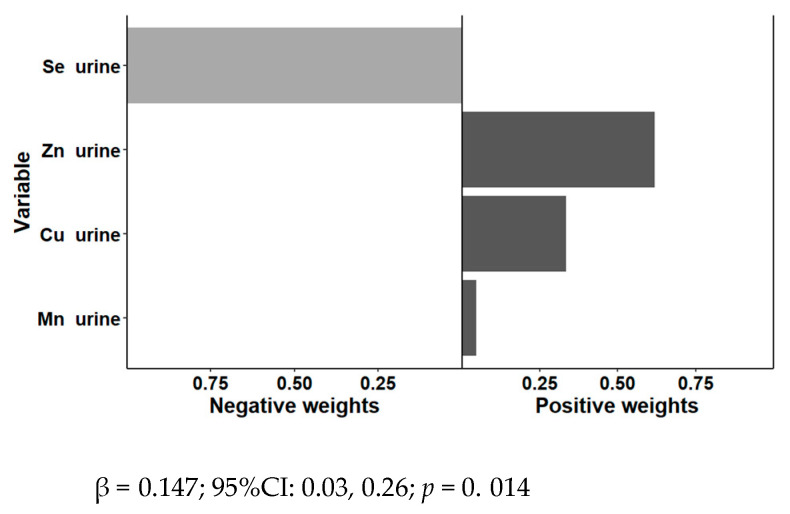
Overall mixture effect estimates (psi1 beta coefficients, 95% confidence intervals, and *p*-values) and the weights of trace elements contributions to the fasting glucose l levels for the combined association with urine Zn, Cu, Se, and Mn. Adjusted model.

Figure 4 shows the overall mixture effect estimates and the weights of trace urinary element contributions to the fasting glucose for fasting glucose in the adjusted model. The weights were positive for Zn, Cu, and Mn. Se presented a negative weight.

Next, using the quantile-based g-computation approach, we assessed the association of the trace elements in urine with diabetes as a categorical variable. The joint effect was associated with an increased probability of having diabetes (OR: 1.91; 95%CI: 1.01–3.92; *p* = 0.046 in the adjusted model 3). Zn, Cu, and Mn had positive weights and Se had a negative weight.

#### 3.4.3. Calculation of TERS for Urine

We calculated the additive scores to summarize the combined influence of trace elements in urine, as given in the Methods section. For category scaling, we first determined the tertiles of urine concentrations for each element (Table S4). On the basis of the individual associations between urine trace elements and cardiovascular risk factors as continuous variables, we computed the directionality of the effect and determined the tertiles associated with an increased risk for cardiovascular risk factors. For each trace element, the tertile with the highest risk was assigned a value of 2, while the tertile with the lowest risk was assigned a value of 0.

The score for the middle tertile was 1. We constructed a global additive TERS for urine (the same TERS for each cardiovascular risk factor) and analyzed the combined influence of trace elements on each cardiovascular risk factor. In this TERS, a direct effect increasing cardiovascular risk was estimated for Zn, Cu, and Se (scored as 0, 1, and 2 for tertile 1, tertile 2, and tertile 3, respectively), and an inverse effect was estimated for Se (2 for tertile 1, 1 for tertile 2, and 0 for tertile 3). Depending on the trace element concentration (low or high), these scores ranged from 0 (lower risk) to 8 (higher risk) for the urine TERS variable for additive combined effect. Then, unadjusted and adjusted general linear or logistic regression models were built for the urine TERS and cardiovascular risk variables. Table S5 displays correlation coefficients and *p*-values for the association between the combined score for Zn, Cu, Se, and Mn in urine and the continuous cardiovascular risk factors. Models 1, 2, and 3 were fitted. We detected a statistically significant association between the combined TERS in urine and fasting plasma glucose (*p* < 0.001) in the same direction and comparable with that obtained earlier using the quantile-based g-computation. However, we obtained additional significant associations of the TERS score with HDL-cholesterol and triglycerides.

### 3.5. Associations between Combined Plasma and Urine Trace Elements with Cardiovascular Risk Factors

**Figure 5:**
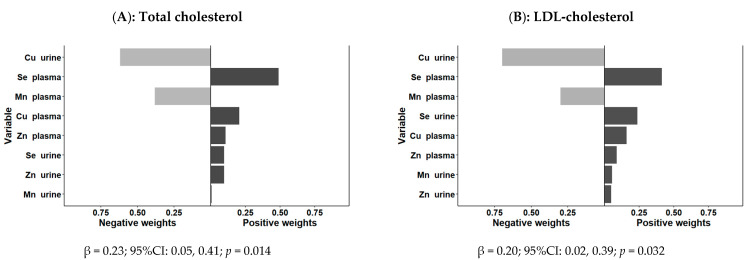
Overall mixture effect estimates and the weights for the plasma and urine trace elements (Zn, Cu, Se, and Mn) (psi1 beta coefficients, 95% confidence intervals (CI), and *p*-values) for the combined association of Zn, Cu, Se, and Mn with total cholesterol (panel **A**) and LDL-cholesterol (panel **B**) as continuous variables. Multivariable adjusted model.

Finally, we explored the joint association of plasma and urine biomarkers for trace elements (Zn, Cu, Se, and Mn) and cardiovascular risk factors. For this combined analysis, we only used the quantile-based g-computation approach. Using the same methodology described in Methods, we used quartiles and estimated the “overall mixture effect from quantile g-computation” (psi1) [103]. Only statistically significant results were obtained for plasma total cholesterol and LDL-cholesterol. Figure 5 shows the association coefficients and the weights for the plasma and urine trace elements and total cholesterol in plasma (panel A) and LDL-cholesterol (panel B) in the model adjusted for sex, age, obesity, and medications.

## 4. Discussion

In this cross-sectional study conducted on a Spanish Mediterranean population aged between 18 and 80 years, statistically significant associations were identified between plasma and/or urine concentrations of essential trace elements (Zn, Cu, Se, and Mn) and highly prevalent cardiovascular risk factors. There is currently much controversy about the protective or risk role of these essential trace elements on cardiovascular risk factors and diseases. This is because studies conducted on this topic over the past few decades have yielded inconsistent results [69,70,71,72,73,74,75,76,77,78,79,80,81,82,83,84,85,86,87,88,89,90,91,92,93,94,95,96,97,98]. Numerous variables [50,55,56,57,58,59,60] can impact the different results of studies in this field. Among these are the characteristics of the population, such as age, gender, the presence of various pathologies, and geographical origin. Additionally, genetic factors can be relevant. Our research was focused on four essential trace elements (Zn, Cu, Se, and Mn) mostly obtained from diet [2]. When comparing the results of different investigations, it is also important to know if the population evaluated had high or low concentrations of the trace elements examined, because depending on these levels, the effects can be different [79,80,81,82,83,84,85,86,87,88,89,90,91]. In our Mediterranean population, the presence of these essential trace elements is relatively high [83,97,113]. Particularly significant is the methodology for evaluating exposure to trace elements from food or other sources [114,115,116]. Considering that the geographical origin influences the trace element content of foods, various biomarkers in biological samples are preferred as more objective measures. However, there is controversy regarding the optimal biomarkers for each trace element [87,88,89,90,91,92,93,94]. We measured the concentrations of Zn, Cu, Se, and Mn in plasma and urine at the same time. This gives us an advantage over other studies that only look at one type of biomarker. Moreover, the majority of previous research has concentrated on the analysis of trace element associations in isolation. However, it is known that the effect of these trace elements can be joint, enhancing or inhibiting each other [95,96,98,99], which is why statistical analyses of association taking into account the combinations of these elements have recently been recommended [101,102]. Several methodological approaches have been proposed for this, and it is advised to utilize a combination of them because there is no consensus regarding the most successful [99]. A number of more traditional investigations have employed principal component analysis [110] for investigating the combined effect of trace elements. However, this approach has several limitations and is being superseded by alternative solutions employing more artificial intelligence algorithms and other methods including shrinkage methods (least absolute shrinkage and selection operator, elastic network model, adaptive elastic-net model), Bayesian kernel machine regression, WQS regression, and quantile-based g-computation [97,100,102,103,111,117,118,119,120]. In our population, we analyzed the associations between trace elements and cardiovascular risk factors using both the single-trace-element approach and the combined-trace element approach. For the combined analysis, we used three approaches including principal component analysis, quantile-based g-computation, and a simple score method so-called TERS. Using quantile-based g-computation, our study is the first to apply the combined analysis of trace elements and their association with cardiovascular risk factors in a Mediterranean population.

Before discussing the obtained results in greater detail, it is necessary to comment on some descriptive aspects of the population’s characteristics and the concentrations of the studied biomarkers. In this Mediterranean population, the mean plasma Zn concentrations are comparable with those observed in a previous study conducted in southern Spain [121] and higher [122,123,124] or lower [125] than those described in other studies. Cu values in this Mediterranean population are slightly higher than those reported in southern Spain [126] and in other populations [124,127]. Mean plasma levels of Se are comparable to previous studies [124,126,128,129]. In the case of Mn, mean plasma values in this Mediterranean population are within the range provided by several authors [130,131,132], higher than those described in some previous studies [124,126,129], and lower than those observed by Shen et al. [128].

In urine, mean Zn values for this population are lower than those reported in the EPIC study [124] in Brazil and [133], but slightly higher than those reported by [134] in women in the United States and in a previous study in Spain [97]. Cu concentrations in the urine of this Mediterranean population are higher than those reported by [124], comparable with those observed by [97], and lower than those found by [133,135,136]. The mean values for urinary Se are higher than those reported by [133,135,136] but lower than those reported by [134]. Likewise, Mn concentrations in urine were lower than those reported by [133,136,137] but comparable with those reported by [134].

When comparing the results of different studies, it is also crucial to examine the demographic features of the population. In our sample, we looked at differences in trace element based on sex and age. In several of the biomarkers studied, we found statistically significant differences between the sexes, but the most relevant were the sex differences in plasma levels of Zn and Cu. These findings are consistent with previous studies [121,126,138,139,140,141]. Regarding urinary concentrations by sex, in our study, we observed that urinary excretion of Zn, Cu, and Se is higher in men. No sex-specific differences were noted for Mn. Once more, no consensus has been found in the literature. In a comprehensive review on urinary excretion of Se, it can be observed that there is a lack of agreement and that urinary Se in men and women shows great variation depending on the study characteristics and the geographical area and content in food [124,142]. Other authors did not observe significant differences in urinary excretion of Se between men and women [143].

The analysis of differences in the plasma concentrations of the trace elements with age revealed that Zn and Cu do not vary significantly in the present study, whereas plasma Se increased and plasma Mn decreased with age. However, the information available in the literature on this subject is contradictory. Some authors reported increases in Zn with age [138], while others report decreases [121,140,141]. In the case of Cu, some authors report increases with age [140,141], similar to Se [126,138,139,140]; however, [144] found very low Se levels in very elderly people. In the case of plasma Mn, our findings agree with those of some researchers [124,140], whereas other authors found increases with age [144,145] or no changes [126]. Regarding changes in urinary content of the trace elements with age, in our case it was found that Zn, Cu, and Se decreased with age, with no significant variations for Mn. Although there is no total agreement on urinary changes of these elements with age, the trend for the excretion of Se to decrease with age has been described by other authors [143] and has been linked to greater likelihood of the malnutrition and organic damage that come with age [146]. However, in a study undertaken in Brazil, a negative correlation between serum concentrations of several minerals and age was reported, but no significant differences were observed in urine levels by age [147]. Due to these potential differences by sex and age, we adjusted the models of the associations with cardiovascular risk factors for these two variables in subsequent statistical analyses to avoid a possible confounding effect.

Since plasma concentrations of Zn, Cu, Se, and Mn are the most widely used and accepted biomarkers for these trace elements [86,87,88,89,90,91,92,93], we began our investigation into the relationship between trace elements and cardiovascular risk factors by analyzing plasma levels of these elements. In this Mediterranean population, correlations between plasma concentrations and urine concentrations of these biomarkers were found to be quite weak. Depending on the biomarker utilized, these low correlations may account for the disparity in results across published studies [48,49,50,51,52,53,54,55,56,57,58,59,60,61,62,63,64,65,66,67,68,69,70,71,72,73,74,75,76,77,78,79,80,81,82,83,84]. Although the majority of published studies analyzed plasma, serum, or blood concentrations, other studies used urine biomarkers [97,124,127,128,129,130,131,132,133]. In the single-trace-element association analysis, we identified a number of statistically significant associations between cardiovascular risk factors and trace elements. Multivariable adjusted models revealed a statistically significant association between plasma Cu and plasma triglycerides, as well as plasma Cu and BMI or waist circumference. However, there was no statistically significant association between plasma Zn levels and the cardiovascular risk factors investigated. No significant associations were obtained for plasma Zn concentrations in the multivariable adjusted model despite the fact that some inverse associations were detected in the unadjusted model. There is currently no agreement on the plasma levels of these elements and their relationship with circulating lipids, blood pressure, fasting glucose, BMI, and/or cardiovascular disease. Rotter et al. [148] positively correlated Zn with circulating triglycerides and Se with total cholesterol, LDL-cholesterol, and triglycerides. Other authors inversely related low circulating levels of Zn with systemic inflammatory activity [80], and with dyslipidemia in the presence of hypertension and hypercholesterolemia [149]. High Cu levels have been linked with hypertriglyceridemia in newborns [150], cardiovascular risk [77], and a positive correlation between Cu levels and total cholesterol and HDL-cholesterol has been described [151]. However, other authors found no correlation between plasma Cu and Zn and lipid parameters [125,152]. Several studies demonstrate a positive correlation between elevated plasma zinc levels and diabetes or glucose at fasting [153,154]. However, in this Mediterranean population, no significant associations were found between plasma Zn levels and these parameters. Other authors found no association between diabetes and the levels of Zn or other trace elements [148,151], even an inverse association with glycated hemoglobin has been reported [155]. Interestingly, despite not detecting statistically significant associations between plasma Zn concentrations and fasting glucose or diabetes risk, we identified a highly significant positive association between urine Zn concentrations and these parameters in our population. Many other studies have found a link between urinary Zn concentrations and glycemia/diabetes [94,155,156,157]. However, the mechanisms underlying this association between Zn concentrations in urine and fasting glucose, but not in plasma, remain unclear. It has been reported that individuals with insulin-dependent diabetes have approximately doubled urinary zinc excretion than controls [158]. This is paradoxical because it has been reported that Zn may help reduce the onset or progression of type 2 diabetes through a variety of mechanisms involving both insulin secretion and peripheral tissue action [159]. However, a suggested mechanism to explain the increased concentrations in urine is that high plasma glucose levels may interfere with the active transport of Zn in renal tubule cells, increasing Zn excretion from the body through urine [160]. However, more research is needed. Additional prospective epidemiological and mechanistic studies should be conducted to better elucidate the associations of Zn plasma and urine levels with fasting glucose and type 2 diabetes risk [161].

We found the most statistically significant and consistent associations between selenium in plasma and plasma lipid concentrations, primarily total cholesterol and LDL-cholesterol concentrations. Despite the fact that selenium was once assumed to be a potent cardiovascular-protective antioxidant with inverse associations with cardiovascular risk factors [79], numerous studies published in populations from the United States, Europe, and even Asia have observed direct associations between higher plasma concentrations of selenium and increases in plasma concentrations of total cholesterol and LDL-cholesterol [51,66,67,68,72,82,162]. Although our results are consistent with observational epidemiological research conducted in the so-called Se replete populations, the mechanism by which greater plasma Se concentrations are linked to hypercholesterolemia remains unclear. It is feasible that a U-shaped link exists between plasma Se concentrations and hypercholesterolemia, in that both low and high Se concentrations would have negative effects on plasma lipids [163,164]. A Se deficit may correlate to a decreased general synthesis of selenoproteins, and this may correspond to an increased oxidative stress and its repercussions in the lipid metabolism (alteration in redox balance, altered protein function, and abnormalities in cardiovascular relevant lipid signaling pathways, among others). Similarly, high plasma Se levels may be associated with the maximal activation of particular selenoproteins, resulting in a compensatory response towards the pro-oxidant effects of Se as well as unfavorable effects in some lipid signaling pathways [164].

In addition to the strong associations between plasma Se and hypercholesterolemia, we detected a statistically significant association between plasma Se and SBP in our Mediterranean population. However, we did not detect significant associations with fasting glucose or diabetes. Other researchers have found associations between plasma Se and blood pressure or diabetes but the findings are inconsistent [67,70,71,79,82]. In the single-trace-element study, plasma Mn was not significantly associated with any cardiovascular risk factor but presented inverse coefficients with many of them. It has been described that the highest Mn serum levels are associated with a lower presence of prediabetes and diabetes in elderly Chinese women but this correlation is found in men when Mn levels are moderate [131].

Regarding BMI and waist circumference, we detected several statistically significant associations with plasma levels in the single-trace-element analysis. However, the associations were in opposite directions (direct associations for plasma Cu and inverse associations for Se). Published studies were also contradictory for plasma trace elements and anthropometric variables, reporting inverse, null, or direct relationships depending on the population [121,126,148,153,154,162,165,166,167,168].

In the combined-trace-element analysis, we used three approaches: a principal component analysis, the quantile-based g-computation, and the construction of a simple score so-called TERS. We conducted the combined analysis with the three approaches separately for plasma and urine and compared the results. Finally, we used the quantile-based g-computation to jointly analyze plasma and urine biomarkers. Quantile-based g-computation is a novel method proposed to specifically address the inherent complexities of high-dimensional mixture data and to estimate the joint effect of the analyzed chemicals [103]. It builds upon previous mixture-based regression models such as weighted quantile sum regression [111]. However, quantile-based g-computation has more advantages, including that does not require a directional homogeneity assumption that all exposures have an effect in the same direction, as modeled in other approaches [102]. Although in the last two years a number of studies employing this approach for the combined analysis of exposure to trace elements have been published [98,99,100,117,118,169], its use is still limited, and there are few published relationships with cardiovascular risk variables to which we can compare our findings. The results of our study’s combined analysis of trace element biomarkers in plasma using quantile-based g-computation were very enlightening. We were able to detect several statistically significant combined associations using this method. Among them is the joint association of the trace elements studied with total cholesterol and LDL-cholesterol concentrations. Perhaps most notably, it has made it possible to easily identify heterogeneity in the associations of different trace elements in combined analyses. We found that none of the cardiovascular risk factors investigated in the combined analysis had all of the trace elements acting in the same direction, increasing the risk. There are elements that, depending on the risk factor studied, have an inverse or direct influence. For example, in the case of total cholesterol, Se, Cu, and Zn all contribute to an increase in risk, whereas Mn decreases it. We were able to validate these findings by using TERS, a new method based on a score of tertiles of trace element concentrations in urine that we developed. In urine, the combined analysis using the quantile-based g-computation approach was very informative regarding the joint exposure effect and yielded statistically significant results for fasting glucose concentrations and diabetes. Again, heterogeneity among the trace elements was detected (Se inversely related and the others directly related). This significant association was also detected in our TERS approach. Finally, we explored the joint association between plasma and urine biomarkers with cardiovascular risk factors and observed statistically significant results for total cholesterol and LDL-cholesterol, revealing a strong association. Given the effects of these plasma lipids and fasting glucose on cardiovascular risk, a better understanding of the individual and combined effects of plasma and urine biomarkers is required for more personalized nutritional advice.

Our research has both strengths and limitations. Among the study’s strengths is that it was conducted on a well-defined general population, which included participants aged 18 to 80 years from a Mediterranean country. In addition, potential confounders have been accounted for in the statistical models. Moreover, we used both a single-trace-element statistical approach and a combined approach to capture the joint influence on the cardiovascular risk variables analyzed. Nonetheless, our study has a number of limitations. The first limitation is that it is a cross-sectional study, from which no causal inferences can be drawn. Similarly, because this is an epidemiological study, we do not analyze the potential mechanisms underlying the statistical relationships identified. Further mechanistic research is required to comprehend the potential mechanisms underlying each statistical association reported. Another limitation is the number of trace materials studied. We investigated Zn, Cu, Se, and Mn; however, it is well known that Fe is another essential trace element that plays a key role in oxidative-stress- and age-related diseases [170,171]. Fe is vital to numerous biological functions, and Fe deficiency or excess can result in a variety of medical conditions related to cardiovascular risk factors or diseases. However, more research is needed at the population level due to the mixed results [172,173,174,175]. It would have been highly interesting to add iron concentration analysis in this Mediterranean population, both for its research as a separate trace element and in the combined joint analysis; therefore, it will be evaluated for further research. Another limitation of the study is that oxidative stress biomarkers have not been determined. Currently, the so-called derivatives-reactive oxygen metabolites (d-ROMs) [176,177,178] are an emerging biomarker of oxidative stress, and their measurement would have offered highly interesting information to relate it to the concentrations of trace elements and to the investigated cardiovascular risk factors. Finally, we did not analyze the genetic factors that may influence cardiovascular risk, which is still another limitation of our study. Several genetic polymorphisms are currently known to be associated with higher concentrations of plasma lipids (i.e., polymorphisms in the APOE gene determining LDL-c levels), blood glucose (i.e., polymorphisms in the TCF7L2 gene), body mass index (i.e., polymorphisms in the FTO gene), and other cardiovascular risk factors [179,180,181,182,183]. More recently, associations of cardiovascular risk factors with microbiota-related polymorphisms have been reported [184]. For all of these reasons, it would have been interesting to investigate if markers of higher or lower genetic risk can modulate the effect of the trace element concentrations on cardiovascular risk phenotypes. A bigger sample size is required for these gene–environment interaction investigations; therefore, we will continue to work in the future.

## 5. Conclusions

In this Mediterranean population, we examined the single and combined association of four essential trace elements (Zn, Cu, Se, and Mn), for which relevant antioxidant effects have been documented, with cardiovascular risk factors, using both the plasma and urine biomarkers. In the single-trace-element analysis, both inverse and direct relationships between these elements and plasma lipids, blood pressure, fasting glucose, or anthropometric factors were observed. The direct associations suggested that larger concentrations may have a pro-oxidant effect increasing some cardiovascular risk factors. However, as this is an observational epidemiological study, no causal or mechanistic conclusions can be drawn. In addition, we examined the effect of joint exposure to trace elements on the cardiovascular risk factors and found some statistically significant joint associations. In plasma, the combined effect of higher plasma trace element levels (mostly Se, Cu, and Zn) was directly related with elevated plasma lipids, whereas the mixture effect in urine (mainly driven by Zn) was primarily associated with plasma glucose. Both parameters are relevant cardiovascular risk factors, suggesting that higher exposures to trace elements should be considered with caution. Nonetheless, in the combined mixture effect analysis utilizing the quantile-based g-computation approach, we identified some trace components in the mixture that were inversely linked with cardiovascular risk variables (i.e., plasma Mn for plasma lipids and urinary Se for fasting glucose). Therefore, additional research employing experimental studies including assessment of genetic factors and oxidative stress biomarkers is necessary to better comprehend the balance or imbalance between trace elements that increase or decrease cardiovascular risk factors.

## Figures and Tables

**Table 1:** General characteristics of the study population by sex.

	Total (n = 484)	Men (n = 160)	Women (n = 324)	*p*
Age (years)	46.28 ± 13.73	45.69 ± 14.75	46.58 ± 13.21	0.520
BMI, kg/m^2^	27.87 ± 5.44	29.17 ± 4.92	27.23 ± 5.58	<0.001
SBP, mmHg	124.81 ± 17.32	132.70 ± 15.88	120.90 ± 16.67	<0.001
DBP, mmHg	78.52 ± 10.87	82.38 ± 12.11	76.60 ± 9.66	<0.001
Total-cholesterol, mg/dL	211.94 ± 40.43	204.78 ± 38.86	215.47 ± 40.78	0.006
LDL-cholesterol, mg/dL	137.82 ± 32.71	137.22 ± 32.21	138.10 ± 32.99	0.781
HDL-cholesterol, mg/dL	59.65 ± 14.13	50.89 ± 11.03	63.94 ± 13.50	<0.001
Triglycerides, mg/dL	108.68 ± 58.15	122.55 ± 66.79	101.85 ± 52.16	<0.001
Fasting glucose, mg/dL	94.91 ± 19.57	99.07 ± 23.08	92.87 ± 17.25	0.003
Creatinine, mg/dL	0.76 ± 0.18	0.94 ± 0.19	0.10 ± 0.01	<0.001
Uric acid, mg/dL	5.31 ± 1.42	6.43 ± 1.25	4.76 ± 1.15	<0.001
Aspartate aminotransferase, U/L	25.31 ± 10.44	29.77 ± 1.13	23.11 ± 7.00	<0.001
Obesity prevalence (%)	32.01	39.62	28.21	0.012
Hypercholesterolemia (%)	35.7	39.9	33.6	0.186
High LDL-cholesterol (%)	35.65	36.09	63.91	0.186
Hypertension (%)	68.13	25.23	74.77	<0.001
Type 2 diabetes (%)	5.41	8.50	3.88	0.039
Antihypertensive drugs (%)	16.85	28.67	11.18	<0.001
Hypolipidemic drugs (%)	14.47	19.33	12.14	0.040
Antidiabetic drugs (%)	3.25	4.67	2.57	0.235
High adherence MD (%) ^1^	49.78	32.29	67.71	0.948
Current smokers %	20.09	16.21	21.90	0.118
Plasma Zinc, µmol/L	15.94 ± 3.45	16.64 ± 3.50	15.59 ± 3.38	0.010
Urine Zinc, µmol/L	5.60 ± 4.26	7.07 ± 4.82	4.87 ± 3.74	<0.001
Plasma Copper, µmol/L	25.59 ± 6.70	21.90 ± 4.23	27.43 ± 6.95	<0.001
Urine Copper, µmol/L	0.13 ± 0.07	0.14 ± 0.067	0.12 ± 0.07	0.042
Plasma Selenium, µmol/L	1.19 ± 0.19	1.21 ± 0.21	1.18 ± 1.87	0.089
Urine Selenium, µmol/L	0.37 ± 0.21	0.42 ± 0.22	0.35 ± 0.20	0.010
Plasma Manganese, nmol/L	74.86 ± 38.40	73.68 ± 40.49	75.45 ± 37.37	0.631
Urine Manganese, nmol/L	7.66 ± 6.30	7.38 ± 3.63	7.80 ± 7.28	0.393

Values are mean ± SE for continuous variables and % for categorical variables; *p*: *p*-value for the comparisons (means or %) between men and women. BMI indicates body mass index. SBP indicates systolic blood pressure. DBP indicates diastolic blood pressure. Obesity prevalence: BMI ≥ 30 kg/m^2^. Hypercholesterolemia: Total-cholesterol ≥ 200 mg/dL or hypolipidemic drugs or high LDL-cholesterol (LDL-cholesterol ≥ 160 mg/dL or drugs). Hypertension: [antihypertensive drug or SBP ≥ 140 mmHg or DBP ≥ 90 mmHg]. Type 2 diabetes: Antidiabetic drug or glucose ≥ 126 mg/dL. ^1^: High adherence to the Mediterranean diet (MD), 9 or more points in the 14-item score.

**Table 2:** Single association between plasma levels of trace elements and cardiovascular risk factors (as continuous).

	Plasma Zn			Plasma Cu		
Variable/Statistic	r (*p*-Value) ^1^	r (*p*-Value) ^2^	r (*p*-Value) ^3^	r (*p*-Value) *^1^*	r (*p*-Value) ^2^	r (*p*-Value) ^3^
Total-cholesterol (mg/dL)	0.014 (0.763)	0.057 (0.245)	0.061 (0.212)	0.120 (0.008)	0.075 (0.124)	0.091 (0.062)
LDL-cholesterol (mg/dL)	0.017 (0.703)	0.029 (0.548)	0.036 (0.465)	0.025 (0.587)	0.015 (0.754)	0.029 (0.559)
HDL-cholesterol (mg/dL)	−0.009 (0.852)	0.111 (0.022)	0.093 (0.062)	0.224 (˂0.001)	0.031 (0.523)	0.091 (0.061)
Triglycerides (mg/dL)	0.009 (0.838)	−0.002 (0.971)	0.034 (0.490)	0.090 (0.048)	0.238 (˂0.001)	0.191 (˂0.001)
SBP (mmHg)	0.063 (0.166)	0.016 (0.748)	0.055 (0.242)	−0.063 (0.172)	0.102 (0.035)	0.073 (0.124)
DBP (mmHg)	0.044 (0.338)	−0.022 (0.654)	−0.014 (0.778)	−0.019 (0.672)	0.116 (0.017)	0.076 (0.107)
Glucose (mg/dL)	−0.049 (0.283)	−0.061 (0.212)	0.026 (0.602)	−0.023 (0.609)	0.049 (0.318)	0.076 (0.120)
BMI (kg/m^2^)	−0.031 (0.498)	−0.077 (0.114)	___	0.059 (0.198)	0.172 (˂0.001)	___
Waist Circumference (cm)	0.010 (0.831)	−0.043 (0.376)	___	−0.045 (0.323)	0.159 (0.001)	___
	**Plasma Se**			**Plasma Mn**		
**Variable**	**r (*p*-value) ^1^**	**r (*p*-value) ^2^**	**r (*p*-value) ^3^**	**r (*p*-value) ^1^**	**r (*p*-value) ^2^**	**r (*p*-value) ^3^**
Total-cholesterol (mg/dL)	0.224 (˂0.001)	0.237 (˂0.001)	0.245 (<0.000)	−0.105 (0.022)	−0.069 (0.156)	−0.029 (0.552)
LDL-cholesterol (mg/dL)	0.194 (˂0.001)	0.191 (˂0.001)	0.209 (<0.001)	−0.108 (0.018)	−0.072 (0.141)	−0.024 (0.624)
HDL-cholesterol (mg/dL)	0.186 (˂0.001)	0.265 (˂0.001)	0.253 (˂0.001)	−0.045 (0.330)	−0.076 (0.120)	−0.073 (0.135)
Triglycerides (mg/dL)	−0.032 (0.484)	−0.065 (0.183)	−0.069 (0.155)	0.013 (0.781)	0.077 (0.116)	0.060 (0.218)
SBP (mmHg)	0.140 (0.002)	0.074 (0.131)	0.103 (0.030)	−0.099 (0.031)	−0.045 (0.361)	−0.054 (0.274)
DBP (mmHg)	0.050 (0.273)	−0.010 (0.841)	0.028 (0.574)	−0.111 (0.016)	−0.058 (0.229)	−0.046 (0.331)
Glucose (mg/dL)	0.036 (0.432)	−0.055 (0.260)	−0.065 (0.183)	0.012 (0.802)	0.096 (0.048)	0.043 (0.378)
BMI (kg/m^2^)	−0.025 (0.578)	−0.105 (0.031)	___	−0.114 (0.013)	−0.058 (0.231)	___
Waist Circumference (cm)	0.011 (0.818)	−0.110 (0.023)	___	−0.096 (0.037)	−0.033 (0.492)	___

Values are correlation coefficients (r) and *p*-values. Trace elements were ln-transformed. ^1^: unadjusted *p*-value; ^2^: *p*-value adjusted by sex and age; ^3^: *p*-value adjusted by sex, age, obesity, and medication when appropriate. r: Pearson; SBP: systolic blood pressure; DBP: diastolic blood pressure; BMI: body mass index.

**Table 3:** Single association between urine levels of trace elements and cardiovascular risk factors (as continuous).

	Urine Zn			Urine Cu		
Variable/Statistic	r (*p*-Value)^1^	r (*p*-Value)^2^	r (*p*-Value)^3^	r (*p*-Value)^1^	r (*p*-Value)^2^	r (*p*-Value)^3^
Total-cholesterol (mg/dL)	−0.060 (0.182)	0.023 (0.639)	0.033 (0.497)	−130 (0.004)	−0.053 (0.277)	−0.053 (0.278)
LDL-cholesterol (mg/dL)	−0.028 (0.535)	0.012 (0.806)	0.026 (0.590)	−0.093 (0.039)	−0.033 (0.498)	−0.036 (0.461)
HDL-cholesterol (mg/dL)	−0.210 (˂0.001)	−0.068 (0.163)	−0.075 (0.123)	−0.153 (0.001)	−0.123 (0.011)	−0.109 (0.026)
Triglycerides (mg/dL)	0.127 (0.005)	0.129 (0.008)	0.120 (0.014)	0.004 (0.922)	0.068 (0.164)	0.042 (0.387)
SBP (mmHg)	0.029 (0.528)	−0.023 (0.644)	−0.007 (0.956)	−0.089 (0.052)	−0.045 (0.357)	−0.038 (0.431)
DBP (mmHg)	0.032 (0.482)	−0.022 (0.654)	−0.017 (0.740)	−0.077 (0.092)	−0.039 (0.418)	−0.048 (0.317)
Glucose (mg/dL)	0.127 (0.005)	0.188 (˂0.001)	0.131 (0.007)	0.039 (0.388)	0.140 (0.004)	0.075 (0.123)
BMI (kg/m^2^)	0.029 (0.523)	0.012 (0.798)	___	−0.037 (0.419)	0.033 (0.503)	___
Waist Circumference (cm)	0.064 (0.157)	0.033 (0.492)	___	−0.044 (0.333)	0.040 (0.407)	___
	**Urine Se**			**Urine Mn**		
**Variable**	**r (*p*-value) ^1^**	**r (*p*-value) ^2^**	**r (*p*-value) ^3^**	**r (*p*-value) ^1^**	**r (*p*-value) ^2^**	**r (*p*-value) ^3^**
Total-cholesterol (mg/dL)	−0.108 (0.017)	0.014 (0.771)	−0.004 (0.933)	0.079 (0.091)	0.054 (0.272)	0.045 (0.358)
LDL-cholesterol (mg/dL)	−0.055 (0.227)	0.047 (0.337)	0.029 (0.555)	0.086 (0.067)	0.058 (0.235)	0.052 (0.285)
HDL-cholesterol (mg/dL)	−0.070 (0.121)	−0.015 (0.757)	−0.022 (0.658)	−0.018(0.706)	−0.001 (0.989)	−0.016 (0.740)
Triglycerides (mg/dL)	−0.129 (0.004)	−0.082 (0.093)	−0.088 (0.070)	0.056 (0.234)	0.019 (0.697)	0.022 (0.652)
SBP (mmHg)	−0.113 (0.013)	−0.071 (0.142)	−0.049 (0.312)	0.114 (0.015)	0.087 (0.074)	0.074 (0.123)
DBP (mmHg)	−0.100 (0.027)	−0.058 (0.236)	−0.062 (0.199)	0.072 (0.123)	0.048 (0.325)	0.036 (0.455)
Glucose (mg/dL)	−0.081 (0.073)	0.012 (0.800)	0.016 (0.743)	0.072 (0.124)	0.029 (0.557)	0.024 (0.628)
BMI (kg/m^2^)	−0.072 (0.110)	0.015 (0.752)	___	0.039 (0.400)	0.009 (0.086)	___
Waist Circumference (cm)	0.064 (0.157)	0.033 (0.492)	___	−0.044 (0.333)	0.040 (0.407)	___

Values are correlation coefficients (r) and *p*-values. Trace elements were ln-transformed. ^1^: unadjusted *p*-value; ^2^: *p*-value adjusted by sex and age; ^3^: *p*-value adjusted by sex, age, obesity, and medication when appropriate. r: Pearson; SBP: systolic blood pressure; DBP: diastolic blood pressure; BMI: body mass index.

**Table 4:** Combined analysis of plasma trace elements. Principal component analysis.

Variable	PC1	PC2	PC3
Eigenvalues	1.315	1.034	1.014
PTV ^1^ (%)	32.9	25.9	25.3
Cumulative PTV (%)	32.9	58.7	84.07
Loadings ^2^ (rotate)			
Zn	0.825	0.207	−0.133
Cu	−0.001	0.031	0.988
Se	0.791	−0.259	0.141
Mn	−0.015	0.965	0.034

PC: Principal component; PTV: Percentage of total variance; ^1^: A varimax rotation was carried out. ^2^: The largest loadings are shown in boldface.

**Table 5:** Tertiles for plasma concentrations of Zn, Cu, Se, and Mn in the studied population.

	T1 Plasma	T2 Plasma	T3 Plasma
Zn (µmol/L)	Lower to 14.78	14.79 to 16.80	16.81 to higher
CU (µmol/L)	Lower to 22.64	22.65 to 26.75	26.76 to higher
Se (µmol/L)	Lower to 1.11	1.12 to 1.25	1.25 to higher
Mn (nmol/L)	Lower to 61.94	61.94 to 88.08	88.08 to higher

T1: Tertile 1; T2: Tertile 2; T3: Tertile 3.

**Table 6:** Combined association between plasma levels of trace elements (Zn, Cu, Se, and Mn) and cardiovascular risk factors (as continuous). Trace elements risk score (TERS) approach.

	TERS Plasma		
	Model 1	Model 2	Model 3
Variable/Statistic	r (*p*-Value) ^1^	r (*p*-Value) ^2^	r (*p*-Value) ^3^
Total-cholesterol (mg/dL)	0.265 (˂0.001)	0.219 (˂0.001)	0.210 (˂0.001)
LDL-cholesterol (mg/dL)	0.214 (˂0.001)	0.178 (˂0.001)	0.173 (˂0.001)
HDL-cholesterol (mg/dL)	0.207 (˂0.001)	0.186(˂0.001)	0.177 (˂0.001)
Triglycerides (mg/dL)	0.036 (0.427)	0.000 (0.994)	0.006 (0.900)
SBP (mmHg)	0.124 (0.006)	0.107 (0.022)	0.110 (0.019)
DBP (mmHg)	0.089 (0.049)	0.063 (0.176)	0.052 (0.256)
Glucose (mg/dL)	0.002 (0.961)	0.064 (0.159)	−0.036 (0.442)
BMI (kg/m^2^)	0.115 (0.011)	0.074 (0.115)	___
Waist Circumference (cm)	0.090 (0.049)	0.063 (0.181)	___

Values are correlation coefficients (r) and *p*-values; ^1^: unadjusted *p*-value; ^2^: *p*-value adjusted by sex and age; ^3^: *p*-value adjusted by sex, age, obesity, and medication when appropriate. r: Pearson coefficient; SBP: systolic blood pressure; DBP: diastolic blood pressure; BMI: body mass index. In the combined TERS analysis, plasma tertiles of Zn, Cu, Se, and Mn were considered and scored (as 0, 1, or 2) for the additive score taking into account the direct or inverse risk effect: Zn, Cu, and Se were scored directly, and Mn was scored inversely.

**Table 7:** Overall combined effect of trace elements (Zn, Cu, Se, and Mn) in urine and cardiovascular risk factors. Based on quantile-g-computation approach.

Cardiovascular Risk Factor	β	95%CI	*p* ^1^
Total-cholesterol (mg/dL)	0.084	0.084, −0.035	0.168
LDL-cholesterol (mg/dL)	0.061	0.061, −0.064	0.340
HDL-cholesterol (mg/dL)	−0.068	−0.068, −0.176	0.222
Triglycerides (mg/dL)	0.031	0.031, −0.089	0.615
SBP (mmHg)	0.022	0.022, −0.086	0.687
DBP (mmHg)	−0.001	−0.001, −0.109	0.986
Glucose (mg/dL)	0.147	0.147, 0.030	0.014
BMI (kg/m^2^) ^2^	0.084	0.084, −0.004	0.061
Waist Circumference (cm) ^2^	0.010	0.010, −0.113	0.872

β: psi1 g-computation coefficient; CI: Confidence interval; ^1^: *p*-value adjusted for sex, age, obesity, and medication when appropriate; ^2^: *p*-value adjusted for sex and age.

## Data Availability

Neither the participants’ consent forms nor ethics approval included permission for open access. However, we follow a controlled data-sharing collaboration model, and data for collaborations will be available upon request pending application and approval. Investigators who are interested in this study can contact the corresponding authors.

## Supplementary Materials

The following supporting information can be downloaded at: https://www.mdpi.com/article/10.3390/antiox11101991/s1, Table S1: Spearman correlation coefficients and *p*-values between plasma and urine concentrations of essential elements (Zn, Cu, Se, and Mn) in a general Mediterranean population (n = 484); Table S2: Single association between plasma levels of trace elements and cardiovascular risk factors (as categories); Table S3: Single association between urine levels of trace elements and cardiovascular risk factors (as categories); Table S4: Tertiles for urine concentrations of Zn, Cu, Se, and Mn in the studied population; Table S5: Combined association between urine levels of trace elements (Zn, Cu, Se, and Mn) and cardiovascular risk factors (as continuous). Trace elements risk score (TERS) approach.
