# Supplementary material for: Single and Combined Associations of Plasma and Urine Essential Trace Elements (Zn, Cu, Se, and Mn) with Cardiovascular Risk Factors in a Mediterranean Population
Source: Antioxidants (Basel). 2022 Oct 7;11(10):1991. doi: 10.3390/antiox11101991 (PMC9598127; doi:10.3390/antiox11101991)
Supplement: Supplementary file 1 [file antioxidants-11-01991-s001.zip › antioxidants-1920958-supplementary-revision.pdf]

Single and combined associations of plasma and urine essential trace elements (Zn, Cu, Se and Mn) with cardiovascular risk factors in a Mediterranean population

Rocío Barragán, Cristina Sánchez-González, Pilar Aranda, José V. Sorlí, Eva M Asensio, Olga Portolés, Carolina Ortega-Azorín, Laura V Villamil, Oscar Coltell, Juan Llopis, Lorenzo Rivas-García, and Dolores Corella

SUPPLEMENTARY MATERIAL

INDEX

Table S1. Spearman correlation coefficients and p-values between plasma and urine concentrations of essential elements (Zn, Cu, Se and Mn) in a general Mediterranean population (n= 484). 2
Table S2. Single association between plasma levels of trace elements and cardiovascular risk factors (as categories). .. 3
Table S3. Single association between urine levels of trace elements and cardiovascular risk factors (as categories). 4
Table S4. Tertiles for urine concentrations of Zn, Cu, Se and Mn in the studied population. 5
Table S5. Combined association between urine levels of trace elements (Zn, Cu, Se and Mn) and cardiovascular risk factors (as continuous). Trace elements risk score (TERS) approach. 5

Table S1. Spearman correlation coefficients and p-values between plasma and urine concentrations of essential elements (Zn, Cu, Se and Mn) in a general Mediterranean population (n= 484).

Essential element	Coefficients	Zn plasma	Cu plasma	Se plasma	Mn plasma	Zn urine	Cu urine	Se urine	Mn urine
Zn plasma	r	1							
	p								
Cu plasma	r	-0.054	1						
	p	0.237							
Se plasma	r	0.310	0.057	1					
	p	<0.001	0.209						
Mn plasma	r	0.051	0.024	-0.119	1				
	p	0.266	0.604	0.009					
Zn urine	r	0.233	-0.107	0.02	-0.029	1			
	p	<0.001	0.018	0.657	0.523				
Cu urine	r	0.037	0.089	-0.082	0.012	0.601	1		
	p	0.416	0.052	0.073	0.794	<0.001			
Se urine	r	0.005	-0.062	0.082	0.025	0.503	0.722	1	
	p	0.917	0.172	0.071	0.586	<0.001	<0.001		
Mn urine	r	0.152	-0.005	0.100	-0.141	0.011	-0.059	0.004	1
	p	0.001	0.910	0.033	0.003	0.814	0.221	0.927	

Correlation coefficient (r: Spearman rho) in the whole population.

Table S2. Single association between plasma levels of trace elements and cardiovascular risk factors (as categories).

Predictor	Zn	p^1	p^2	p^3	Cu	p^1	p^2	p^3	Se	p^1	p^2	p^3	Mn	p^1	p^2	p^3
**Sex**																
Men	16.64±3.49	0.010	<0.001	<0.001	21.89±4.23	<0.001	<0.001	<0.001	1.21±0.21	0.089	0.063	0.132	73.68±40.48	0.631	0353	0.161
Women	15.58±3.38				27.44±6.95				1.18±0.19				75.45±37.37			
**Age**																
18-42 years	16.31±3.33				26.13±8.86				1.17±0.19				82.70±37.63			
43-54 years	15.57±2.88	0.090	0.236	0.318	25.78±5.30	0.858	0.895	0.456	1.20±0.18	0.064	0.003	0.004	68.08±37.10	0.005	0.006	0.001
55-80 years	16.18±3.86				25.33±6.54				1.21±0.16				77.26±39.08			
**Hypercholesterolemia**																
LDL-c ≥160 mg/dL	15.85±2.84				25.44±5.78				1.21±0.18				72.12±38.89			
LDL-c <160 mg/dL	16.02±4.27	0.84	0.030	0.104	25.79±8.13	0.582	0.186	0.505	1.15±0.19	<0.001	<0.001	<0.001	79.78±37.13	0.593	0.410	0.965
**Diabetes**																
Diabetic	15.18±2.99				23.86±5.31				1.17±0.23				77.58±40.40			
Non-diabetic	16.05±3.46	0.113	0.075	0.282	25.67±6.81	0.301	0.903	0.484	1.19±0.19	0.905	0.509	0.470	75.78±38.25	0.776	0.357	0.394
**Hypertension**																
Yes HBP	16.05±2.87				25.12±5.72				1.20±0.21				73.50±38.13			
No HBP	15.92±3.70	0.762	0.840	0.687	25.77±7.12	0.426	0.050	0.279	1.18±0.19	0.304	0.515	0.647	76.74±30.02	0.507	0.529	0.609
**Waist circumference**																
No risk	5.44±4.54	0.790	0.821	0.167	0.12±0.07	0.073	0.022	0.498	0.33±0.18	0.908	0.114	0.485	7.93±6.39	0.169	0.728	0.927
Risk	5.69±3.94				0.13±0.08				0.41±0.22				7.20±6.11			

Values are mean±standard error (SE); Zn: Zinc. Cu: Copper. Se: Selenium. Mn: Manganese; Units are expressed as μmol/L for Zn, Cu and Se, and nmol/L for Mn; [1]: Unadjusted model. [2]: Model adjusted for sex and age. [3]: Model adjusted for sex, age, obesity and medications. LDL-c: LDL-cholesterol. HBP: High blood pressure.

Table S3. Single association between urine levels of trace elements and cardiovascular risk factors (as categories).

Predictor	Zn	p^1	p^2	p^3	Cu	p^1	p^2	p^3	Se	p^1	p^2	p^3	Mn	p^1	p^2	p^3
**Sex**																
Men	7.07±4.82	<0.001	<0.001	<0.001	0.14±0.07	0.04	0.006	0.003	0.42±0.22	0.010	<0.001	<0.001	7.38±3.62	0.393	0.393	0.382
Women	4.87±3.74				0.12±0.07				0.35±0.20				7.79±7.27			
**Age**																
18-42 years	6.82±5.14				0.15±0.08				0.46±0.22				6.67±4.19			
43-54 years	5.15±3.55	<0.001	0.001	0.012	0.11±0.06	<0.001	<0.001	<0.001	0.35±0.19	<0.001	<0.001	<0.001	8.08±8.04	0.055	0.229	0.490
55-80 years	4.63±3.45				0.11±0.06				0.28±0.16				7.87±5.59			
**Hypercholesterolemia**																
LDL-c ≥160 mg/dL	5.66±4.35	0.717	0.251	0.106	0.12±0.07	0.007	0.805	0.747	0.36±0.21	<0.001	0.231	0.862	7.83±5.40	0.217	0.749	0.644
LDL-c <160 mg/dL	5.50±4.09				0.13±0.07				0.39±0.21				7.74±7.72			
**Diabetes**																
Diabetic	7.47±3.64	<0.001	0.001	0.036	0.14±0.08	0.534	0.107	0.175	0.32±0.18	0.175	0.733	0.682	10.14±12.63	0.128	0.274	0.141
Non-diabetic	5.52±4.27				0.13±0.08				0.37±0.21				7.25±5.22			
**Hypertension**																
Yes HBP	5.50±3.68	0.781	0.793	0.399	0.12±0.05	0.105	0.783	0.949	0.32±0.18	0.002	0.085	0.605	8.22±6.24	0.121	0.583	0.799
No HBP	5.56±4.43				0.13±0.07				0.39±0.21				7.25±6.20			
**Waist circumference**																
No risk	5.44±4.54	0.696	0.225		0.12±0.07	0.039	0.856		0.33±0.18	<0.001	0.068		7.93±6.39	0.280		0.976
Risk	5.69±3.94				0.13±0.08				0.41±0.22				7.20±6.11			

Values are mean±standard error (SE). Zn: Zinc. Cu: Copper. Se: Selenium. Mn: Manganese; Units are expressed as µmol/L for Zn, Cu and Se, and nmol/L for Mn; [1]: Unadjusted model. [2]: Model adjusted for sex and age. [3]: Model adjusted for sex, age, obesity and medications. LDL-c: LDL-cholesterol. HBP: High blood pressure.

Table S4. Tertiles for urine concentrations of Zn, Cu, Se and Mn in the studied population.

	T1 urine	T2 urine	T3 urine
Zn (μmol/L)	Lower to 3.27	3.28 to 6.13	6.13 to higher
Cu (μmol/L)	Lower to 0.089	0.09 to 0.14	0.15 to higher
Se (μmol/L)	Lower to 0.25	0.25 to 0.43	0.44 to higher
Mn (nmol/L)	Lower to 6.37	6.38 to 8.19	8.20 to higher

T1: Tertile 1; T2: Tertile 2; T3: Tertile 3.

Table S5. Combined association between urine levels of trace elements (Zn, Cu, Se and Mn) and cardiovascular risk factors (as continuous). Trace elements risk score (TERS) approach.

	TERS urine		
	**Model 1**	**Model 2**	**Model 3**
**Variable**	r (p-value)[1]	r (p-value)[2]	r (p-value)[3]
Total-cholesterol (mg/dL)	0.039 (0.393)	0.043 (0.347)	0.052 (0.267)
LDL-cholesterol (mg/dl)	0.069 (0.127)	0.063 (0.167)	0.074 (0.116)
HDL-cholesterol (mg/dL)	-0.183 (<0.001)	-0.157 (0.001)	-0.154 (0.001)
Triglycerides (mg/dL)	0.180 (<0.001)	0.191 (<0.001)	0.190 (<0.001)
SBP (mmHg)	0.154 (0.001)	0.111 (0.018)	0.082 (0.082)
DBP (mmHg)	0.108 (0.018)	0.085 (0.069)	0.060 (0.201)
Glucose (mg/dL)	0.139 (0.002)	0.216 (<0.001)	0.172 (<0.001)
BMI (kg/m^2)	0.119 (0.009)	0.080 (0.087)	___
Waist Circumference (cm)	0.154 (0.001)	0.079 (0.094)	___

Values are correlation coefficients (r) and p-values; [1]: unadjusted *p* value; [2]: *p* value adjusted by sex and age; [3]: *p* value adjusted by sex, age, obesity and medication when appropriate. r: Pearson; SBP: systolic blood pressure; DBP: diastolic blood pressure; BMI: Body Mass Index; In the combined TERS analysis, urine tertiles of Zn, Cu, Se and Mn were considered and scored (as 0, 1 or 2) for the additive score taking into account the direct or inverse risk effect: Zn, Cu and Mn were scored directly, and Se was scored inversely.
